# Groundwater Irrigation and Arsenic Speciation in Rice in Cambodia

**DOI:** 10.5696/2156-9614-8.19.180911

**Published:** 2018-09-10

**Authors:** Tom Murphy, Kongkea Phan, Emmanuel Yumvihoze, Kim Irvine, Ken Wilson, David Lean, Borey Ty, Alexander Poulain, Brian Laird, Laurie Hing Man Chan

**Affiliations:** 1 International University, Phnom Penh, Cambodia; 2 University of Ottawa, Canada; 3 Nanyang Technological University, Singapore; 4 Texas State University, San Marcos, Texas, USA; 5 Lean Environmental, Apsley, Ontario, Canada; 6 Institute of Technology Cambodia, Phnom Penh; 7 University of Waterloo, Canada

**Keywords:** arsenic, remediation, bioaccumulation, irrigation, dimethylarsinic acid, rice

## Abstract

**Background.:**

Arsenic bioaccumulation in rice is a global concern affecting food security and public health.

**Objective.:**

The present study examined arsenic species in rice in Cambodia to characterize health risks with rice consumption and to clarify uncertainties with Codex guidelines.

**Methods.:**

The present study collected 61 well water samples, 105 rice samples, 70 soil samples, and conducted interviews with 44 families in Preak Russey near the Bassac River and Kandal Province along the Mekong River in Cambodia. Analyses of metals, total arsenic and arsenic species were conducted in laboratories in Canada, Cambodia and Singapore.

**Results.:**

Unlike in Bangladesh, rice with the highest total arsenic concentrations in Cambodia contains mostly organic arsenic, dimethylarsinic acid (DMA), which is unregulated and much less toxic than inorganic arsenic. The present study found that storing surface runoff in ditches prior to irrigation can significantly reduce the arsenic concentration in rice. It is possible to remove > 95% of arsenic from groundwater prior to irrigation with natural reactions.

**Conclusions.:**

The provision of high quality drinking water in 2015 to Preak Russey removed about 95% of the dietary inorganic arsenic exposure. The extremes in arsenic toxicity that are still obvious in these farmers should become less common. Rice from the site with the highest documented levels of arsenic in soils and water in Cambodia passes current Codex guidelines for arsenic.

**Informed Consent.:**

Obtained

**Competing Interests.:**

The authors declare no competing financial interests.

## Introduction

Arsenic bioaccumulation into rice is a major global concern affecting food security and public health. In parts of Bangladesh, arsenic bioaccumulation and resulting diseases are the greatest cause of mortality. Graziano stated in 2010 that one in five deaths in Bangladesh was related to arsenic.^1^ The primary source of arsenic in Bangladesh, Cambodia, and several other countries in South and Southeast Asia is arsenopyrite oxidation, which naturally occurs in rocks in the Himalayan Mountains.^2^

This process releases arsenic into solution which binds to sediments in the Mekong River which then flows from the Himalayan Mountains to form soils in Cambodia. Natural biogeochemical and hydrologic processes can be enhanced by human activities to dissolve this naturally occurring arsenic in soils.^3^,^4^ Decades ago, tens of thousands of wells were dug in Cambodia to reduce diarrhea, cholera, and other diseases initiated by drinking water from surface sources. From 2002 to 2015, the number of deaths from diarrhea decreased by 55%.^5^ However, childhood mortality remains high in part because only 53% of Cambodians have “safe” drinking water.^6^ In 1999, high concentrations of arsenic were found in Cambodian groundwater and by 2006, the first cases of arsenicosis in Cambodia were reported.^7^ As with bacterial contamination of water, management of arsenic in water needs improvement. The country standard for arsenic in drinking water is 50 μg/L^8^, whereas the World Health Organization guideline is 10 μg/L.^9^ Initially, Cambodia had few resources capable of measuring 10 μg/L of arsenic and the risks associated with bacterial contamination were considered to be more serious than 50 μg/L of arsenic.^8^ Both of these problems remain but have been improved. Currently, many farmers believe that if their wells have no more than 50 μg/L of arsenic, they do not have to collect rainwater or take other steps to reduce their arsenic intake. Moreover, some wells are also used for irrigation which results in bioaccumulation of arsenic into rice.^10^

There are over 2.4 million people living in the arsenic-contaminated zone of Cambodia.^11^ In the most affected areas, people try to reduce their intake of arsenic, but most of the farmers in these areas consume water and food from their land on a daily basis. Over 100,000 people are at high risk of chronic arsenic exposure.^7^ Documented effects can be as subtle as impairment of intellectual development in children or extreme, including amputations of limbs to remove cancerous growths.^12^,^13^ Congenital birth defects and mortality from arsenic are also well known. The most serious source of arsenic toxicity is from drinking groundwater rich in arsenic. In some of the most contaminated sites, safe drinking water is now being provided. However, secondary sources of arsenic, such as arsenic that has bioaccumulated in crops are also a concern. In areas of Bangladesh with good drinking water, consumption of rice with more than 200 μg/kg of arsenic is associated with significantly higher levels of cancer.^14^ Some rice in the present study site has twice this threshold for cancer induction, while in comparison some rice in India and Bangladesh contains five times this threshold for cancer induction.^10^,^15^ Irrigation with groundwater is more extensive and has been used over twice as long in Bangladesh compared to Cambodia. The high concentration of inorganic arsenic in rice in South and Southeast Asia has been called a “health emergency”.^16^

**Abbreviations**
*DMA*: Dimethylyarsinic acid
*IDRC*: International Development Research Centre
*TMA*: Trimethyl arsine
*XRF*: X-ray fluorescence

There is an urgent need to improve the cultivation of rice in Cambodia and other countries with similar geochemical characteristics to avoid the high levels of arsenic bioaccumulation in rice found in Bangladesh and parts of India. These new approaches should be effective, but also sensitive to local conditions in developing countries. Farmers in the main research site in the present study are concerned about their health and rarely eat rice that they grow with groundwater. However, some farmers became very concerned that our research could interfere with their ability to sell rice. While Cambodia has a very similar environment to Bangladesh, irrigation with groundwater has not taken place nearly as long or extensively in Cambodia as it has in Bangladesh. The objectives of the present study are to characterize the concentrations of all major arsenic species in rice collected from different sites in Cambodia that used three irrigation methods, conduct interviews with farmers on their farming practices and to explore potential changes in irrigation methods to reduce arsenic concentrations in rice.

## Methods

**Figure 1:**
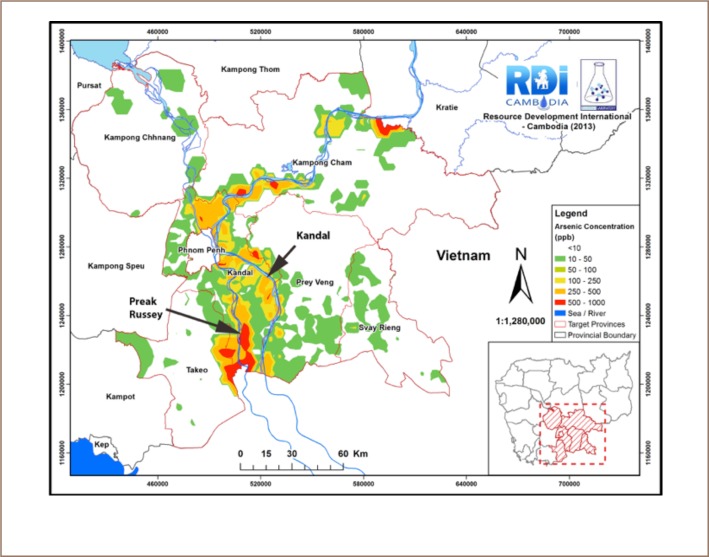
Primary sampling sites

Figure 1 shows the study area and was prepared by Resource Development International from more than 10,000 analyses of wells.^7^ The arsenic content of the groundwater varies from extremes in Preak Russey on the Bassac River to low to moderate levels of arsenic in groundwater near the Mekong River in a control site we called Kandal. Both sites are in many ways very similar. The Bassac River is a distributary of the Mekong River. Both sites are mostly flood plains using very similar agricultural processes. Details of the study sites between the Mekong and Bassac rivers, sample collection and processing can be found in our International Development Research Centre, Canada (IDRC) report.^17^ Unless otherwise stated, all analyses of water and rice samples were conducted in a laboratory at the University of Ottawa. Trace elements in the water samples including arsenic were measured by inductively coupled plasma–mass spectrometry (ICPMS) according to the US EPA Method 200.^18^ Arsenic species including As (III), As (V), MMA, and DMA were quantified using the method developed by Agilent Technologies.^19^ The coefficient of variation of seven duplicate sets of rice samples was 3.6±2.3%. The results of NIST 1568b certified reference brown rice and DORM-2 and DORM-4 certified reference fish samples were within 20% of expected results. Efforts were made to collect rice at every site where soil or water samples were collected and interviews were conducted. Interviews were conducted with 44 families in Preak Russey village and a second less contaminated site in Kandal Province in Cambodia. Earlier studies conducted by Resource Development International Cambodia (RDIC) were used to select the highly contaminated farms in Preak Russey and less contaminated farms along the Mekong River that comprised the Kandal site.^7^ An interview form was developed using preliminary visits to these farms in 2014 and 2015. The form was designed to quantify aspects of the farming methods and to facilitate communications and collaboration with the farmers (*Supplemental Material*). The first question to each farmer obtained informed consent. In total, 102 rice samples in husks (paddy rice) were collected. After air drying, the husks were removed from paddy rice by hand. When possible, polished rice and rice bran were also collected.

### Site details

About one fourth of the farmers in the Preak Russey study area used drainage ditches to remove excess water at the end of the rainy season. By chance, a farmer was discovered pumping groundwater from his well, first into a ditch and then later onto his rice field (sample site Preak Russey-4) in the belief that this process would cleanse the water. The ditch was 200 m long. Sampling took place about 180 m from the pump. Another farmer was discovered by chance that first pumped groundwater from a well into a ditch prior to irrigating his rice paddy (site Preak Russey-1) that was about 80 m away. Later, a third farmer (site Preak Russey-10) used the insights from this study (as discussed below) and adopted this strategy of pumping groundwater from his well into a ditch prior to irrigation of his fields. His ditch was >500 m long, but sampling occurred in the first 100 m only. These ditches were about 4 m wide and up to 2 m deep. The farmers added no chemicals to the irrigation water. No aeration was used, except for naturally occurring oxidation. The only physical action that occurred was the introduction of nets to impede fish from entering the water pumps, but the nets also acted as a physical surface for periphyton growth and aided establishment of a layer of floating aquatic plants (mostly water hyacinth, Eichhornia crassipes). The floating plants impeded transport of floating orange scum to the pumps. Furthermore, no efforts were made to remove brush from the ditches which also acted as substrate for periphyton growth and impeded transport of floating scum to the pumps. Water samples were collected once from the ditches of Preak Russey-4 and once from Preak Russey-10. Sample collection was restricted by the timing of irrigation, weather and our awareness of the situation. Once the rains had begun, well water in the ditches was diluted to unknown amounts. Sampling was avoided if rain had occurred the week before a visit. When the rains became vigorous, irrigation stopped.

The floating orange scum was collected and freeze dried at the Fisheries Administration, Ministry of Agriculture, Forestry and Fisheries, Cambodia. Metals were determined in the scum by X-ray fluorescence (XRF) analysis using the Camcontrol XRF analyzer (Niton XL3tGOLDD) and certified reference standards from Thermo Fisher Scientific.^17^ Statistical analyses used Excel and VassarStats.^20^

## Results

**Figure 2:**
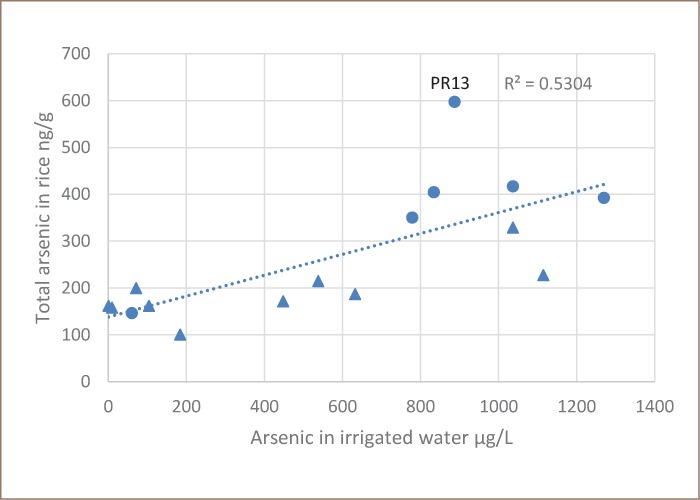
Total arsenic in rice versus total arsenic in irrigation water PR13 is Preak Russey-13. The • are farms where rice was collected after July 1 2016. The triangles represent farms where rice was collected before June 1.

The average total arsenic content of rice and water (field wells) in Preak Russey was 315±150 μg/kg and 959±351 μg/L vs. 158±33 μg/kg and 65±51 μg/L, respectively, in Kandal (Table 2, Appendix 2 in the IDRC report).^17^ Figure 2 shows a significant relationship (p> 0.01) between the content of arsenic in irrigation water and the total arsenic content of rice collected in 2016. The data set that is plotted in Figure 2 lacks paired data from some farms, because at times farmers declined to provide rice samples. The rice with the most arsenic contamination was from site Preak Russey-13, a farm without a ditch for irrigation with surface water. With the Preak Russey-13 data removed, the r^2^ improved from 0.530 to 0.681. Rice from site Preak Russey-13 contained as much as six times the total arsenic as rice in farms with the lowest concentrations. The total arsenic level from site Preak Russey-13 was 598 μg/kg, which is very close to levels reported by Phan et al. in the worst samples from Preak Russey (578 μg/kg).^10^ For Preak Russey-13's second crop of rice, the farmer could only use groundwater for irrigation. When farmers have a choice, as they do early in the season, they use surface water, as they are aware of the problem with arsenic in groundwater. A t-test indicates that this comparison of samples is significant (p = 0.0045). There are other variables involved, but this indicates that the effect of using surface water rather than groundwater with >1000 μg/L of arsenic is important. There was one matched pair of samples. Site Preak Russey-2 has a ditch; the first crop (March 19, 2016) of rice had 329 μg/kg of total arsenic, while the second crop (July 26, 2016) with mostly groundwater irrigation had 417 μg/kg of total arsenic. There are other aspects of irrigation that illustrate the importance of avoiding use of groundwater. The total arsenic content of rice grown at site Preak Russey-4 (187 μg/kg) was the second lowest observed at Preak Russey; site Preak Russey-4 is where the farmer used a treatment ditch which effectively removed 99% of the arsenic (Table 1, Appendix 2 in the IDRC report, and as discussed below).^17^ In Preak Russey, rice with the lowest total arsenic content (147 μg/kg, Preak Russey-15) was always grown completely with surface water.

**Figure 3:**
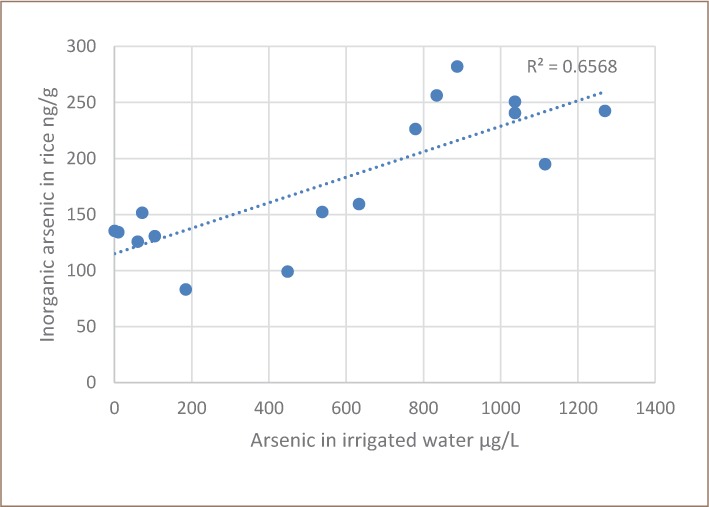
Inorganic arsenic in rice versus total arsenic in irrigation water

**Figure 4:**
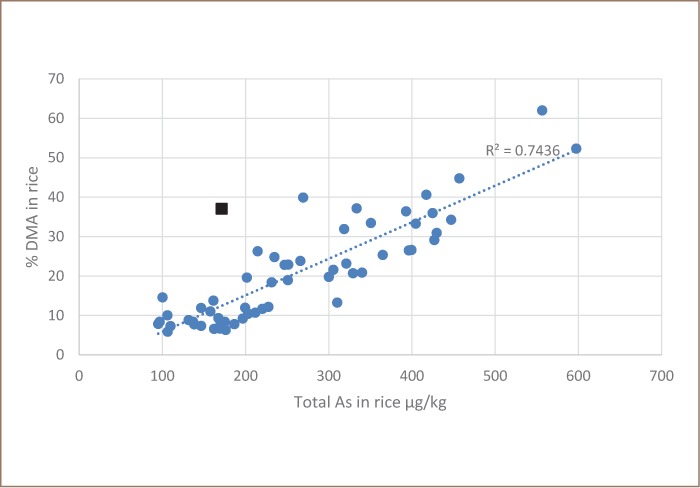
Rice dimethylarsinic acid content of rice versus total arsenic in rice The square represents the only sample of the IR09 rice variety.

The concentration of inorganic arsenic in rice was lower than the total arsenic in rice and the correlation between total arsenic in water and inorganic arsenic in rice was stronger (*Figure 3*). The reason for the differences between Figure 2 and Figure 3 is the presence of organic arsenic, dimethylarsinic acid (DMA). The primary knowledge gained from this project was that unlike in Bangladesh, rice with the highest total arsenic in Cambodia contains mostly organic arsenic, DMA (*Figure 4*).

Speciation of arsenic in 14 irrigation wells indicated that >99% of the arsenic was inorganic. The arsenic in well water used to fill a small pond initially was 88% arsenite and 12% arsenate; after a 5-minute residence time in the pond, the arsenic in the pond outlet was 95% arsenate (*Appendix 1*).^17^ The arsenic was highly reactive and bioavailable.

## Discussion

Our results show that the rice with the highest total arsenic in Cambodia had mostly organic arsenic, DMA (*Figure 4*). Dimethylarsinic acid has 1/66th the toxicity (minimal risk levels) of inorganic arsenic and DMA is not regulated by Codex, the global guidelines for rice export established by the Food and Agriculture Organization of the United Nations and World Health Organization (FAO/WHO).^21–23^ This information greatly reduces the probability of rice exported from Cambodia failing Codex guidelines.

The 2014 Codex guidelines require that if the total arsenic content of polished rice exceeds the preliminary guideline of 300 μg/kg, then polished rice must be processed with speciation analysis and have less than 200 μg/kg of inorganic arsenic. The present study mainly sampled brown rice, i.e. not yet polished. Brown rice stores much longer, so it is the most common rice for farmers. All samples of brown rice in the present study would pass the new Codex guideline of 350 μg/kg of inorganic arsenic for brown rice.^22^ Codex uses the term husked rice for brown rice. Most of the farmers did not have polished rice. However, we were able to collect nine samples of both polished and brown rice from eight farms. On average, polished rice contained 70.6±13% of the inorganic arsenic content of brown rice (Table 3, Appendix 2 in the IDRC report).^17^ Duxbury observed a 30% reduction of the inorganic content of 23 varieties of boro rice in Bangladesh brown rice milled to remove 10% of the rice mass to produce white rice.^24^ This was consistent in all varieties measured by Duxbury and is similar to the results of the present study.

When this proportion of inorganic to total arsenic (0.71) is multiplied by the inorganic content of our brown rice samples, it is predicted that none of the polished rice in Preak Russey would exceed the Codex guidelines. Significantly, this calculation results in rice from site Preak Russey-13 being within 1% of the Codex guidelines. This calculation is only an estimation and does not take into account the greater content of DMA in rice with higher total arsenic. The only polished rice sample collected from a farm with high total arsenic, Preak Russey-2, contained 138 μg/kg inorganic arsenic (Table 4, Appendix 2 in the IDRC report)^17^ and would pass the more stringent Chinese guideline discussed by Meharg et al.^25^ The ratio of inorganic arsenic to total arsenic in site Preak Russey-2 polished rice is lower than other polished rice (0.55, Table 4, Appendix 2 in the IDRC report).^17^ Rice bran in Preak Russey was rich in inorganic arsenic (494±119 μg/kg, n=9) and is used as fish and animal feed (Table 5, Appendix 2 in the IDRC report).^17^ More data on polished rice and rice bran are needed from farms in Preak Russey with high levels of total arsenic.

Significantly, the two sample sites with rice with the highest total inorganic arsenic, Preak Russey-2 and Preak Russey-13, had high concentrations of DMA or cacodylic acid in rice (170 and 306 μg/kg, respectively). The replication on duplicates for the DMA content of Preak Russey-13 was excellent (313 and 299 μg/kg). Because DMA is organic arsenic, at present the Preak Russey-13 rice meets export guidelines.

The variability of arsenic levels in rice between farms is large and it is possible for a polished rice sample to fail the current Codex or Chinese guidelines. Without improvements in arsenic management, farms that continue to extensively use groundwater for irrigation could result in rice at Preak Russey that exceeds Codex guidelines. Mixing of rice from sites like Preak Russey-13 site with other farms could alleviate the risk of blocked sales, but the best strategy would be to avoid using groundwater irrigation as much as possible. Building ditches for rainwater storage is an effective initial strategy and readily fits into the need for drainage at the end of the rainy season as well. Water treatment for groundwater irrigation should also be considered.

### Arsenic removal in irrigation

The arsenic in irrigation wells was highly reactive and bioavailable. Most publications on irrigation with groundwater stress chemical reactions such as adsorption of arsenic to iron in soil.^26^,^27^ Microbial reactions in soils and the treatment ditch warrant further analysis. The bio-oxidation of arsenite may yield useable energy or may form part of a detoxification process.^28^

It is possible for the genomes of microbes in the floating scum to be determined, which could validate the process of microbial bio-oxidation of arsenite.^29^,^30^ Furthermore, the composition of the gas produced in the floating scum might provide insights into bio-oxidation and the mass balance of arsenic. Studies in Bangladesh suggest that the slow build-up of arsenic in soils relative to the application input rate from irrigation suggests that most of the arsenic from irrigation is removed in the rainy season.^26^,^27^ This likely is a very important flux of arsenic, but it is possible that some of the gas produced in the treatment ditch or fields is trimethyl arsine (TMA) gas which could be vented to the atmosphere.^31–37^

**Figure 5:**
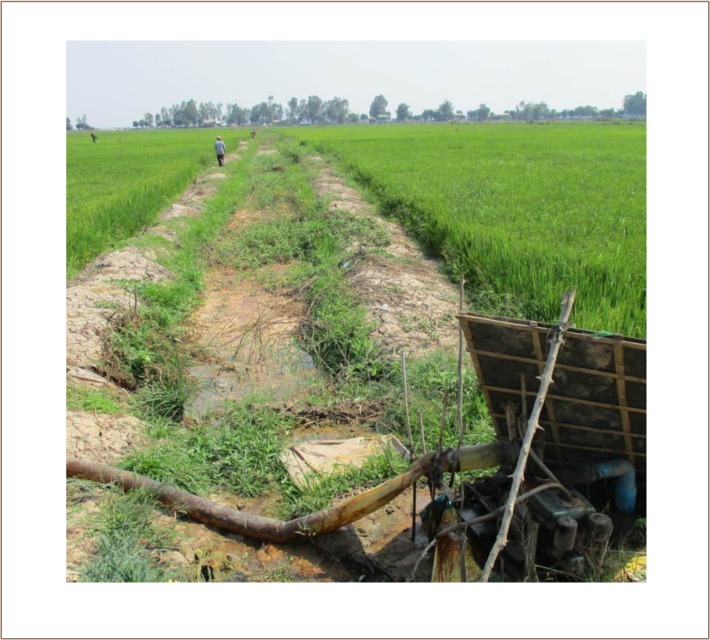
Treatment ditch Preak Russey-4

Iron can be readily seen floating in flocs in the paddy fields and treatment ditches (*Figure 5*). The floc floats because of gas production. The mean iron and arsenic content of this freeze-dried floc was 26,000±239 and 125±8 mg/kg, respectively (n=3). The ratio of iron to arsenic in the floc from Preak Russey-10 was 208/1, whereas the ratio of iron to arsenic in the Preak Russey-10 well was 16/1 (well water 16742 μg/L iron and 1044 μg/L arsenic). The enrichment of iron in the floc was substantial. Using the ratio of iron to arsenic in the well water and multiplying it by iron in the floc, either 87% of the arsenic volatilized (likely as trimethyl arsine, TMA) or an enriched arsenic precipitant formed and was not observed. The XRF analysis of arsenic and iron at these high concentrations is quite reliable (Appendix 3 in the IDRC report).^17^ The most likely explanation is volatilization of TMA and direct analysis of the gases collected over the treatment ditch should be considered.

Further evaluation of the effectiveness of a treatment ditch in removing arsenic is needed. Further analysis was not possible due to funding and project duration constraints. Based on the results of the present study, farmers were advised not to use groundwater, so they rebuilt an irrigation ditch to access water from the Bassac River. There was no funding to redirect their efforts and the dry season of 2016–2017 was wet enough that farmers did not need to irrigate with groundwater. Some aspects of the treatment ditch would best be evaluated in a controlled experiment on a farm that was rented for at least a three-year period. Evaluation of the toxicity of irrigation water compared to treated water could only be performed in such an experimental design. Likewise, the effects of irrigation water on arsenic bioaccumulation in rice varieties and soil is best carried out with control over all aspects of farming procedures. The cost of land rental, technical direction and field help is minor compared to the potential significance of continuing to produce rice with arsenic. Long-term monitoring is required to assess any potential accumulation of arsenic in ditches and mobilization of arsenic into fields.

Most farmers understand that ditches improve storage/access to surface water and produce rice with lower arsenic concentrations. Education and management are needed, along with an increase in the number of ditches. Furthermore, although treatment ditches effectively remove arsenic, to optimize their use, the mechanism of arsenic removal must be fully understood. The likely mechanism of volatilization of TMA could be evaluated with silver nitrate traps. Gas trapping of TMA should be evaluated in treatment ditches, rice paddies and in air near rice paddies. Likely TMA production is substantial in the rice paddies and optimization of TMA production in treatment ditches does not change volatilization of arsenic in the general area. Dilution of TMA is likely considerable, but assessment of ambient air near the rice paddies could help manage health concerns through appropriate risk assessment of relevant exposures, including combustion of rice husks and rice stubble.

Although even the most contaminated rice from the study area is legal for export, there remains a growing concern about arsenic in rice. In 2014, Dr. Andy Meharg stated that “standards need to be set to protect those most at risk and 50 ppb for children and 100 ppb for all rice products would be achievable with concerted effort of regulators and industry, though - as every dose of inorganic arsenic carries a risk - the lower the better.”^38^ On January 1, 2016, the standard for arsenic in children's food in the European Union was reduced to 100 ppb and subsequently has been effectively managed.^39^ By comparison, there is no equivalent arsenic standard for children in Asia. One farmer in Preak Russey stated that arsenic is responsible for his 10-year old child being unable to sit up without support since birth. He drives more than 5 hours from his rice farm to buy rice. This is anecdotal evidence, but consistent with arsenic exposure found in this area and documentation on other health issues such as amputations of cancerous limbs caused by arsenic in this area of Cambodia.^13^,^40^ Using the same intake calculation method as was applied to a study area 40 km from Preak Russey, but substituting the inorganic arsenic content of rice from Preak Russey (mean of 11 farms 202.5±42 μg/kg, 45 samples) and the mean inorganic arsenic content in water from Preak Russey (23 wells 883±332 μg/L), this calculation indicates that the provision of high quality drinking water in 2015 to Preak Russey removed about 95% of the dietary (inorganic) arsenic exposure.^41^ Prior to 2015, drinking water was a 23-times greater source of inorganic arsenic than rice. The current dietary intake of arsenic at Preak Russey could be more accurately measured, but the importance of the new water supply would not change significantly. These extremes in arsenic toxicity that are still obvious in farmers in Preak Russey should become less common. Further clarification may be best performed by analysis of current level of arsenic in blood and urine in farmers in Preak Russey, with associated health analysis and interviews. In this region, it is important to better characterize the risk of arsenic exposure from rice consumption by conducting arsenic speciation analysis on a more routine basis. Dietary advice or regulation should be based on the concentrations of specific arsenic species. It is important to educate farmers on irrigation methods that can reduce arsenic accumulation in rice. Risk communication, especially with regard to water consumption is also of vital importance.

## Conclusions

Unlike in Bangladesh, rice with the highest total arsenic concentrations in Cambodia contained mostly organic arsenic, DMA, which is unregulated and much less toxic than inorganic arsenic. Analysis of arsenic species in a greater variety of rice in Cambodia is necessary to characterize the health risk of consumption and for regulation purposes. Storing surface runoff or groundwater in ditches prior to irrigation can significantly reduce the arsenic concentration in rice. Properly designed ditches or holding ponds can remove > 95% arsenic from groundwater prior to irrigation. The mechanism of arsenic removal should be resolved to facilitate treatment optimization.
