# Supplementary material for: Groundwater Irrigation and Arsenic Speciation in Rice in Cambodia
Source: J Health Pollut. 2018 Sep 10;8(19):180911. doi: 10.5696/2156-9614-8.19.180911 (PMC6257176; doi:10.5696/2156-9614-8.19.180911)
Supplement: Supplementary file 1 [file hapn-8-19-180911_s01.pdf]

Supplemental Material

The Interview Form

Rice Cropping Practices – IDRC As Bioaccumulation in Rice Project

The purpose of this survey is to provide us with an understanding about current agricultural practices in the communities along the Mekong River. Information you provide will only be viewed by the study team, is confidential, and cannot be used to identify any individual.

Date of Survey: ________________________________

Survey Team: _______________

GPS Readings of well: Easting Northing
Phone

Socio-economic Information
First, we will ask you a few questions so that we understand something about your life here:

1. Are you head of household? ☐ yes ☐ no
2. Age: ________ Male female
3. How many people live in this household? _________________________________
4. What is your main occupation (source of income)? _________
5. Do you have other sources of income? _________

Agricultural Information
Next, we will ask you a few questions about your agricultural practices:

6. What crops do you grow? _____
7. How much land do you farm (response in ha is preferable)? ________
8. If you grow rice:

a. How many crops do you get each year? ______________________

i. Crop 1: Plant what month?____________________________

Crop 1: Harvest what month? _________________

Crop 1: Rice type grown (check one):

☐ "504" (Vietnam)
☐ "OM" (Vietnam)
☐ "Sen Krahob" (Cambodia)

☐ Other __________________

Crop 1: Yield per ha?

Crop 1: Selling price per ha? ___ _________

ii. Crop 2: Plant what month? _____________

Crop 2: Harvest what month? ___________________

Crop 2: Rice type grown (check one):

☐ "504" (Vietnam)
☐ "OM" (Vietnam)
☐ "Sen Krahob" (Cambodia)

☐ Other ______________________________________

Crop 2: Yield per ha? __________________________________

Crop 2: Selling price per ha? ____________________________

b. Do you irrigate your rice? Yes No
i. If yes, where does the water come from? _______
ii. How a much does it cost to irrigate one crop?______
iii. How many years have you irrigated? ____________
iv. What is the capacity of your pump? _______
v. Diameter of well head piping (cm) _____
vi. How often do you pump (# of days per week)? ____
vii. How long do you pump each time (# of hours each time). __
viii. Can you estimate how much water you use for each crop?
c. What do you do with your rice? Eat it only Sell it only Both eat and sell

9. Do you use fertilizers? Yes No
a. If yes, what type? (photo each, and circle all appropriate)
NPK Urea DAP Manure
i. If NPK is used, NPK ratio _________
ii. Brand(s), countries of origin:
NPK: ____________________
Urea: ________________________
DAP: ____
Manure: ____
b. If yes, how much fertilizer do you use for one crop (kg)?
NPK: ________
Urea: ___________________________
DAP: __________________________
Manure: ____
c. If yes, how much does the fertilizer cost?
NPK:
Urea: ______
DAP: ____
Manure: ____
d. If yes, where did you learn about fertilizers? ___trial and error

10. Do you have any knowledge about the System of Rice Intensification (SRI) method to grow rice?
11. Have you tried using SRI on your farm?
12. Have you ever tried growing corn as your second crop? ☐ yes ☐ no
If the answer in Q. 12 is no, why not?

13. If the answer in Q. 12 is yes:
a. What was the yield per ha? ______________________________
b. What was the selling price per ha? __________

Knowledge on Arsenic
Finally, we will ask you about your home water use and understanding of arsenic issues.

14. Do you cook with groundwater? ☐ yes ☐ no (if no, what do you use?)

15. Do you drink groundwater? ☐ yes ☐ no (if no, what do you use?)
16. What do you know about arsenic?
Comments:
